# Contrasting effects of human settlement on the interaction among sympatric apex carnivores

**DOI:** 10.1098/rspb.2021.2681

**Published:** 2022-04-27

**Authors:** Ugyen Penjor, Christos Astaras, Samuel A. Cushman, Żaneta Kaszta, David W. Macdonald

**Affiliations:** ^1^ Wildlife Conservation Research Unit, The Recanati-Kaplan Centre, Tubney House, Abingdon Road, Oxford OX13 5QL, UK; ^2^ Nature Conservation Division, Department of Forests and Park Services, Thimphu, Bhutan; ^3^ Forest Research Institute, ELGO-DIMITRA, Thessaloniki, Greece; ^4^ USDA, Rocky Mountain Research Station, 2500 S, Pine Knoll Dr, Flagstaff, AZ 86001, USA

**Keywords:** eastern Himalaya, human settlement, interspecific interaction, large carnivores, multi-species occupancy model

## Abstract

In the face of a growing human footprint, understanding interactions among threatened large carnivores is fundamental to effectively mitigating anthropogenic threats and managing species. Using data from a large-scale camera trap survey, we investigated the effects of environmental and anthropogenic variables on the interspecific interaction of a carnivore guild comprising of tiger, leopard and dhole in Bhutan. We demonstrate the complex effects of human settlement density on large carnivore interactions. Specifically, we demonstrate that leopard–dhole co-occupancy probability was higher in areas with higher human settlement density. The opposite was true for tiger–leopard co-occupancy probability, but it was positively affected by large prey (gaur) abundance. These findings suggest that multi-carnivore communities across land-use gradients are spatially structured and mediated also by human presence and/or the availability of natural prey. Our findings show that space-use patterns are driven by a combination of the behavioural mechanism of each species and its interactions with competing species. The duality of the effect of settlement density on species interactions suggests that the benefits of exploiting anthropogenic environments are a trade-off between ecological opportunity (food subsidies or easy prey) and the risk of escalating conflict with humans.

## 1. Introduction

Interactions among species are the foundations of the structure and integrity of ecological communities [1]. The carnivore guild is mainly shaped by competition and intraguild predation [2–4]. Large carnivores regulate trophic levels by controlling prey populations and by moderating mesocarnivore populations and their effects, thus indirectly affecting both herbivore and plant communities [5,6]. However, their ecological roles are compromised by anthropogenic-induced habitat loss and fragmentation, prey depletion, and mortality due to direct persecution [7,8]. Studies have shown that extirpation of apex predators from an ecosystem leads to an increase in ungulate populations, which in turn results in overgrazing and suppression of plant growth and ultimately degradation of habitat for a range of species [9]. Assessing the impact of anthropogenic and environmental changes on large carnivore communities is key to understanding broader community structure and resilience across the human land-use gradient. Yet, the most recent studies on the interaction among threatened large carnivores have been predominantly confined to small, protected reserves (a few 100 km^2^) [10–12]. Inferring about carnivore community interactions, beyond protected areas, at a landscape scale to better understand carnivore community structure and stability is surprisingly rare.

According to coexistence theory, competing species must segregate at least along one or more dimensions of their ecological niches in order to coexist [13,14]. When such niche differentiation is achieved, interspecific competition is reduced and a greater number of species can coexist [15]. Resource competition intensifies when two or more species occupy a similar ecological niche and hence vie to exclude each other [16]. According to intraguild predation theory, the distribution of dominant predators is influenced by food availability, whereas that of subordinate species is determined by food availability and safety from predation [4]. Hence, from this theory, it is apparent that resource productivity underpins large carnivore interactions. While coexistence stabilizes ecosystem structure, lack of it leads to the population decline or extirpation of one of the interacting species, which can have rippling effects across the trophic level [9]. Antagonistic interactions among carnivores (i.e. competition and predation) change the species' spatial and temporal ecology [17,18]. One of the ways that large carnivores share prey and habitat is by spatial and temporal partitioning [19,20]. Food availability, it has been argued, is a principal driver of carnivore spatial organization and thus guild structure [21]. Where resources are rich and dispersed, sharing among carnivores is possible without additional cost to each species’ fitness [22].

One of the central tenets of community ecology is to understand interactions among species and their abiotic environments, as a way of predicting species' geographical distributions and abundances [23]. Anthropogenic influences alter species interactions by benefiting some and disadvantaging others [24]. Studies have demonstrated that a decrease in large/apex carnivore presence benefits mesopredators [25]—a phenomenon known as mesocarnivore release—resulting in the decline of smaller prey populations [26] and the further intensification of competition among smaller carnivores. Although species sensitive to humans avoid encounters by altering their spatio-temporal behaviour or habitat use [27], human tolerant species can modify their behaviour to increase overlap with humans in order to reduce intraguild competition [28]. However, such behaviour comes at a heightened risk of conflict with humans [29]. Furthermore, when human disturbances result in reduced carnivore diversity, it may lead to loss of ecological services if the prospering species are unable to perform the ecological functions of the extirpated ones [30], with detrimental consequences for ecosystem resilience [31].

The tigers (*Panthera tigris*), leopards (*Panthera pardus*) and dholes (*Cuon alpinus*) are syntopic across most of their range in the south and southeastern Asia and play an important role in regulating prey populations and balancing trophic levels. They are all categorized as ‘Threatened’ on the International Union for Conservation of Nature (IUCN) Red List due to decreasing population trends and are therefore considered high-priority conservation species across their ranges [32]. They prefer prey of a similar size range and hence intraguild competition among them is likely high [33]. This study aims to assess the patterns of coexistence and evaluate factors mediating sympatry among the large carnivore guilds, beyond what has been already studied within protected areas. Our primary objectives were: (1) to investigate the effects of anthropogenic and environmental variables on the occurrence of large carnivores across human land-use gradient and (2) to examine the influence of human disturbance on spatial interaction of threatened large carnivores at site and landscape level. Given the previously reported sensitivity of many large carnivores to human disturbance, we predicted that all three species will be negatively affected by human disturbance and that interaction between species pairs (tiger–leopard, tiger–dhole and leopard–dhole) will be negatively affected by settlement density and disturbance at the site. We also tested the hypothesis that prey choice and prey abundance would be able to support multiple carnivores and facilitate sympatry [12]. Our study is specifically aimed at unravelling the spatial partitioning mechanism of large carnivores across human land-use gradient and its implications on the management of threatened large carnivores.

## 2. Material and methods

### (a) Study area

Our study area was Bhutan, a small country of 38 394 km^2^ area, landlocked between the Tibetan Autonomous Region (China) to the north and India to the east, west and south (electronic supplementary material, appendix S1). About 70% of the country is covered by forests. The elevational gradient increases northwards from 100 m to 7000 m, mediating the vegetation composition: subtropical southern foothills are characterized by broadleaved forests, the temperate zone by cool-broadleaved and conifer mix forests, and the alpine and subalpine zones by rhododendron-scrub mix forests and stunted shrubs. The topography is typically rugged terrain with deep gorges, narrow valleys and steep slopes. The mean annual temperature ranges between 10 and 24°C and annual precipitation between 300 and 6000 mm. Human population density is low: 20 people km^−2^ [34]. Bhutan is entirely within the eastern Himalayan biodiversity hotspot [35] and harbours a rich spectrum of wildlife, including charismatic megafauna such as tigers, leopards, dholes, snow leopards (*Panthera uncia*), clouded leopards (*Neofelis nebulosa*) and Asian elephants (*Elephas maximas*) among others. More than half of the country's area is part of the national protected area network.

### (b) Camera trap survey

We conducted a camera trap survey following a regular square-grid design based on a putative female tiger home range of 25 km^2^ to guide the placement of camera traps [36]. We placed a pair of infrared motion-triggered camera traps in each grid cell along human or animal trails (except in the absence of trails, when cameras were placed randomly) at a height of approximately 45 cm above the ground with a 1 s reset time and burst image mode (for maximum captures). A total of 1129 unbaited camera stations were established. The camera models used were Bushnell, Cuddeback, HCO-Scoutguard, Reconyx, U-Way and Panthera. The mean distance between camera traps was 2.9 km (s.d. = 1.2 km) but this distance was highly variable depending on the terrain and site accessibility. We omitted grid cells that contained dense urban areas (more than 70% of the grid space), and those above 4500 m altitude due to the low probability of capturing tigers, which was the primary objective of the survey. For logistical convenience, the country was divided into two blocks, and camera traps were deployed during the dry season when most sites were easier to access. We deployed camera traps in the south block for 141 days (between March 2014 and June 2014) and in the north for 157 days (between October 2014 and March 2015). We monitored camera stations every 30th day to retrieve data, change batteries, replace memory cards and clear the camera's field of view of vegetation.

### (c) Model covariates

We modelled variation in occupancy probability using covariates that addressed our hypotheses: human settlement density, forest cover, disturbance at the camera trap site, river density, slope and prey abundance. We calculated settlement density using a point shapefile of all known households obtained from the Forest Department. After rasterizing the number of households at 90 m pixel resolution, we calculated the mean number of households among all pixels within a 4 km radius from each camera site in ArcGIS [37]. This variable reflects the density of houses per pixel (90 m) and when averaged across the 4 km radius characterizes human-related influence at the landscape scale. Moreover, as this variable is highly correlated with other infrastructures such as farm roads and highways, it also serves as a proxy for built-up areas. We used global tree cover data (rescaled to 90 m resolution) and averaged over a 4 km radius [38] for each camera site. For river density, we rasterized the river shapefile (obtained from the forest department of Bhutan) at a pixel resolution of 90 m and calculated the per cent of river pixels within a 4 km radius of each camera station. The radius of 4 km was selected to represent the mean home range of the smallest species of the three carnivores (i.e. leopard's average home range size of approx. 50 km^2^) [39]. Studies have shown that large prey species were the most preferred prey of tigers, leopards and dholes, accounting for a significant portion of the total prey biomass [33,40–42]. We used muntjac (*Muntiacus muntjak*), gaur (*Bos gaurus*) and serow (*Capricornis thar*) relative abundance as prey covariates in all large carnivore models. First, we used a hierarchical N-mixture model [43] to estimate the relative abundance of prey from camera trap data as a function of forest cover, elevation (extracted from digital elevation model) [44] and settlement density while accounting for imperfect detection probability using the ‘unmarked’ package [45] in R [46]. We then used site-level abundance as prey covariates in occupancy models of each species. The mean daily encounter rates (independent captures 30 min apart) of humans and livestock (dogs, cattle and horses) were used as a covariate to represent the effect of disturbance (hereafter human disturbance) at the site level (camera trap site). The detection covariates were trail (coded as 1 for cameras placed on-trail and 0 otherwise), human disturbance and trap effort (number of days a camera trap was functional during the survey) to account for unequal sampling effort (83.3[1–156]). For details on the mean and variation of covariates, see electronic supplementary material, appendix S2. We controlled for the geographical variation of north and south blocks on occupancy by including blocks as a random effect in the marginal occupancy model.

### (d) Multispecies interaction model

We used the multispecies species occupancy model [47] to examine the interaction among large carnivores. This model extends the interaction model of [48] but accommodates the effects of covariates and does not need *a priori* assumption of asymmetric interactions (i.e. need not consider one species dominant over the other). It models the interaction between two or more species using a multivariate Bernoulli (MVB) distribution, $Z_{i}\;∼\;\mathrm{MVB}(\psi_{i})$ where Z_i_ is a three-dimensional vector of binary detection/non-detection data denoting latent occupancy state of all three study species and *ψ*_i_ is a 2^3^-dimensional vector that denotes the probability of all possible states Z_i_ can take [47]. For the three species, we modelled Z as, $Z\;∼\;\mathrm{Categorical}(\psi_{000},\;\psi_{100},\;\psi_{010},\;\psi_{001},\;\psi_{110},\;\psi_{011},\;\psi_{101},\;\psi_{111})$. Here, the latent occupancy state for all species present is represented as *ψ*_111_, when all are absent as *ψ*_000_, when either two are present as *ψ*_110_, *ψ*_011_, *ψ*_101_ and only one is present as *ψ*_001_, *ψ*_100_, *ψ*_010_. As in any occupancy model, *ψ* can be modelled as a function of covariates and it describes the probability a site is occupied by only one (first order), two (second order) or more (higher order) interacting species. The latent states for S species have 2^S^−1 possible combinations described by natural parameters (*f*) (*sensu* [47]) which describe the log-odds a species occupies a site. For three species, the natural parameters are *f*_1_, *f*_2_, *f*_3_, *f*_12_, *f*_13_, *f*_23_, *f*_123_. Fixing *f*_12_, *f*_13_, *f*_23_ and *f*_123_ to zero assumes independence in species occurrence. We modelled marginal occupancy (i.e. *f*_1_, *f*_2_, *f*_3_ or no interaction) as a function of settlement density, forest cover, human disturbance, river density, slope, and prey abundance and conditional occupancy (i.e. *f*_12_, *f*_13_, *f*_23_ or pairwise interaction) as a function of settlement density, forest cover, disturbance, prey and interaction between prey and settlement density. We did not model higher-order interaction (i.e. *f*_123_ = 0) because we assumed the probability that three species occurred together was purely a function of species-specific and pairwise interaction parameters.

We binned detection/non-detection data for each camera station into 15-day per sampling occasion replicates to increase temporal independence of detections and reduce overdispersion. We built a set of 19 candidate models to test our hypotheses regarding the effects of anthropogenic and environmental variables on the occupancy of each species and interspecific interactions (table 1). We compared and ranked the models based on the Watanabe–Akaike information criterion (WAIC) [49] and selected the model with the lowest WAIC value for interpretation. We assessed model fit by comparing observed data to simulated data using Freeman–Tukey discrepancy and computed Bayesian *p*-value as a summary of posterior predictive check [50]. All covariates were *z*-standardized prior to analysis. We fitted models in JAGS [51] called through R using the package jagsUI [52]. We used uniform priors on first-order occupancy and detection intercepts and weakly informative normal priors on first- and second-order occupancy and detection slopes (see Data availability statement). We ran the models with three parallel chains of 100 000 Markov chain Monte Carlo (MCMC) iterations each, discarding 50 000 iterations during adaptation and 30 000 in burn-in phases and retaining every 50th (thinning) posterior sample for inference. Model convergence was diagnosed using the Gelman–Rubin statistic ($\hat{R}$) for all parameters and visual inspection of trace plots [53]. All parameters in our model achieved convergence ($\hat{R}<1.1$). The top-rank model adequately fitted our data (Bayesian *p*-value = 0.13; electronic supplementary material, appendix S4).

**Table 1.** Model selection results. *β*_0_ = marginal occupancy intercept; *β_x_* = marginal occupancy slopes; *γ*_0_ = two-way interaction intercept; *γ_x_* = two-way interaction slopes. 0 = no interaction; 1 = constant two-way interaction (intercept only). WAIC = Watanabe–Akaike information criterion. ΔWAIC = delta WAIC (difference between WAIC of the top and subsequent models). Prey = gaur, muntjac, and serow.

marginal occupancy	conditional occupancy	detection	WAIC	ΔWAIC
*β*_0_ + *β*_1_ settlement + *β*_2_ disturbance + *β*_3_ forest + *β*_4_ river + *β*_5_ slope + $\overset{X=3}{\underset{x=0}{\sum }}\beta_{x}$ prey	*γ*_0_ + *γ*_1_ settlement + *γ*_2_ disturbance + $\overset{X=3}{\underset{x=0}{\sum }}\gamma_{x}$ prey	*α*_0_ + *α*_1_ trail + *α*_2_ effort + *α*_3_ disturbance	3585.2	0
*β*_0_ + *β*_1_ settlement + *β*_2_ disturbance + *β*_3_ forest + *β*_4_ river + *β*_5_ slope + $\overset{X=3}{\underset{x=0}{\sum }}\beta_{x}$ prey	1	*α*_0_ + *α*_1_ trail + *α*_2_ effort + *α*_3_ disturbance	3590.8	5.6
*β*_0_ + *β*_1_ settlement + *β*_2_ disturbance + *β*_3_ forest + *β*_4_ river + *β*_5_ slope + $\overset{X=3}{\underset{x=0}{\sum }}\beta_{x}$ prey	0	*α*_0_ + *α*_1_ trail + *α*_2_ effort + *α*_3_ disturbance	3592.6	7.4
*β*_0_ + *β*_1_ settlement + *β*_2_ disturbance + *β*_3_ forest + *β*_4_ river + *β*_5_ slope + $\overset{X=3}{\underset{x=0}{\sum }}\beta_{x}$ prey	*γ*_0_ + *γ*_1_ settlement + *γ*_2_ disturbance + $\overset{X=3}{\underset{x=0}{\sum }}\gamma_{x}$ prey + *γ_i_* prey × settlement	*α*_0_ + *α*_1_ trail + *α*_2_ effort + *α*_3_ disturbance	3596.2	11
*β*_0_ + *β*_1_ settlement + *β*_2_ disturbance + *β*_3_ forest + *β*_4_ river + *β*_5_ slope + $\overset{X=3}{\underset{x=0}{\sum }}\beta_{x}$ prey + *β_i_* prey × settlement	*γ*_0_ + *γ*_1_ settlement + *γ*_2_ disturbance + $\overset{X=3}{\underset{x=0}{\sum }}\gamma_{x}$ prey + *γ_i_* prey × settlement	*α*_0_ + *α*_1_ trail + *α*_2_ effort + *α*_3_ disturbance	3617.8	32.6
*β*_0_ + *β*_1_ settlement + *β*_2_ disturbance + *β*_3_ forest + *β*_4_ river + *β*_5_ slope + $\overset{X=3}{\underset{x=0}{\sum }}\beta_{x}$ prey	*γ*_0_ + *γ*_1_ settlement + *γ*_2_ disturbance	*α*_0_ + *α*_1_ trail + *α*_2_ effort + *α*_3_ disturbance	3621.9	36.7
*β*_0_ + *β*_1_ settlement + *β*_2_ forest + $\overset{X=3}{\underset{x=0}{\sum }}\beta_{x}$ prey	*γ*_0_ + *γ*_1_ settlement	*α*_0_ + *α*_1_ trail + *α*_2_ disturbance	4221.1	635.9
*β*_0_ + *β*_1_ forest + $\overset{X=3}{\underset{x=0}{\sum }}\beta_{x}$ prey	*γ*_0_ + *γ*_1_ settlement	*α*_0_ + *α*_1_ trail + *α*_2_ disturbance	4226.6	641.4
*β*_0_ + *β*_1_ settlement + *β*_2_ forest + $\overset{X=3}{\underset{x=0}{\sum }}\beta_{x}$ prey	1	*α*_0_ + *α*_1_ trail + *α*_2_ disturbance	4229.7	644.5
*β*_0_ + *β*_1_ settlement + $\overset{X=3}{\underset{x=0}{\sum }}\beta_{x}$ prey	*γ*_0_ + *γ*_1_ settlement	*α*_0_ + *α*_1_ trail + *α*_2_ disturbance	4237.7	652.5
*β*_0_ + *β*_1_ settlement + *β*_2_ forest + $\overset{X=3}{\underset{x=0}{\sum }}\beta_{x}$ prey	0	*α*_0_ + *α*_1_ trail + *α*_2_ disturbance	4238.7	653.5
*β*_0_ + *β*_1_ forest + $\overset{X=3}{\underset{x=0}{\sum }}\beta_{x}$ prey	1	*α*_0_ + *α*_1_ trail + *α*_2_ disturbance	4240.8	655.6
*β*_0_ + *β*_1_ settlement + $\overset{X=3}{\underset{x=0}{\sum }}\beta_{x}$ prey	1	*α*_0_ + *α*_1_ trail + *α*_2_ disturbance	4245.2	660
*β*_0_ + *β*_1_ forest + $\overset{X=3}{\underset{x=0}{\sum }}\beta_{x}$ prey	0	*α*_0_ + *α*_1_ trail + *α*_2_ disturbance	4247.1	661.9
*β*_0_ + *β*_1_ settlement + $\overset{X=3}{\underset{x=0}{\sum }}\beta_{x}$ prey	0	*α*_0_ + *α*_1_ trail + *α*_2_ disturbance	4256.8	671.6
*β*_0_ + *β*_1_ settlement + *β*_2_ forest	*γ*_0_ + *γ*_1_ settlement	*α*_0_ + *α*_1_ trail + *α*_2_ disturbance	4283.6	698.4
*β*_0_ + *β*_1_ settlement + *β*_2_ forest	1	*α*_0_ + *α*_1_ trail + *α*_2_ disturbance	4294.2	709
*β*_0_ + *β*_1_ settlement + *β*_2_ forest	0	*α*_0_ + *α*_1_ trail + *α*_2_ disturbance	4306.6	721.4
*β*_0_	0	0	4344.8	759.6

## 3. Results

Across the total survey effort of 73 259 sampling (trap) days at 849 stations (out of 1129, the rest were lost to animal vandalism, theft and malfunction) in 2014 and 2015, we obtained 323, 497 and 421 detections of tigers, leopards and dholes, respectively, at 151, 197 and 210 sites, respectively. The top model consisted of all pairwise interactions between species as a function of settlement density, human disturbance, and prey, and performed significantly better than models with constant (intercept only) pairwise interaction or independent occurrence (no interaction, table 1). The marginal occupancy parameters in the top model were settlement density, human disturbance, forest cover, river density, slope and prey (gaur, muntjac and serow) (table 1).

The detection probability of all species was positively associated with cameras placed on-trail than off-trail and trap effort. Human disturbance at the camera site had a strong negative effect on the detection probability of all three species (electronic supplementary material, appendices S11–S13).

**Figure 1.**
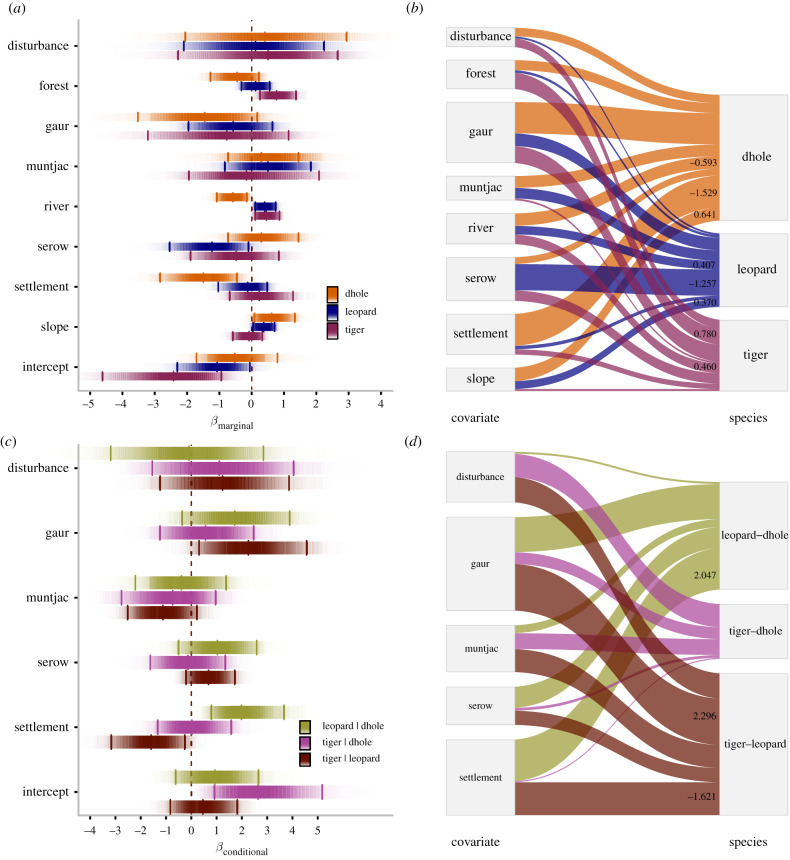
The effects of anthropogenic and environmental variables on individual species (*a*,*b*) and pairwise interactions (*c*,*d*). The posterior density plots (*a*,*c*) show $\hat{\beta }$-coefficients shaded in proportion to the posterior probability density (dark shade = high density). Effect sizes are on the logit scale and represent the effect of 1 s.d. change in covariate value. Lines indicate median and 95% credible intervals. (*b*,*d*) The relationship between covariates and species occupancy probability. Line width corresponds to the strength of the relationship. Significant relationships are illustrated with coefficient estimates. (Online version in colour.)

**Figure 2.**
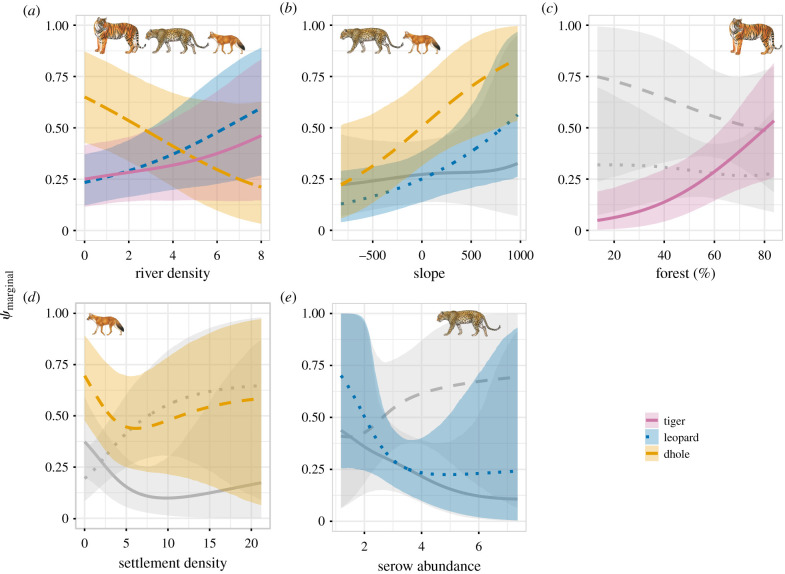
Marginal occupancy probability of the tiger (orchid solid line), leopard (blue dotted line) and dhole (gold dashed line) as a function of river density, slope, forest cover, settlement density and prey (serow) abundance, mean response and associated 95% credible interval are represented by lines and shaded ribbons, respectively. Light grey colour indicates a non-significant relationship (95% CRI straddles zero). (Online version in colour.)

The density of river within 4 km buffer was strongly and positively associated with marginal occupancy probability of tiger (*β* [95% credible intervals] = 0.46[0.11 – 0.86]) and leopard (0.41[0.11 – 0.74]) but significantly negatively with that of dhole (−0.59[−1.08 − –0.15]) (figure 1). Slope had a strong positive effect on marginal occupancy of leopard (0.37[0.04 – 0.71]) and dhole (0.64[0.09 – 1.33]) (figure 2). Only tiger showed a strong positive association with forest cover (0.78[0.26 – 1.37]) (figure 2). Only dhole and leopard were strongly and negatively associated with settlement density (−1.53[−2.83 − –0.46]) and serow abundance (−1.26[−2.54 − –0.10]), respectively (electronic supplementary material, appendices S5–S7).

**Figure 3.**
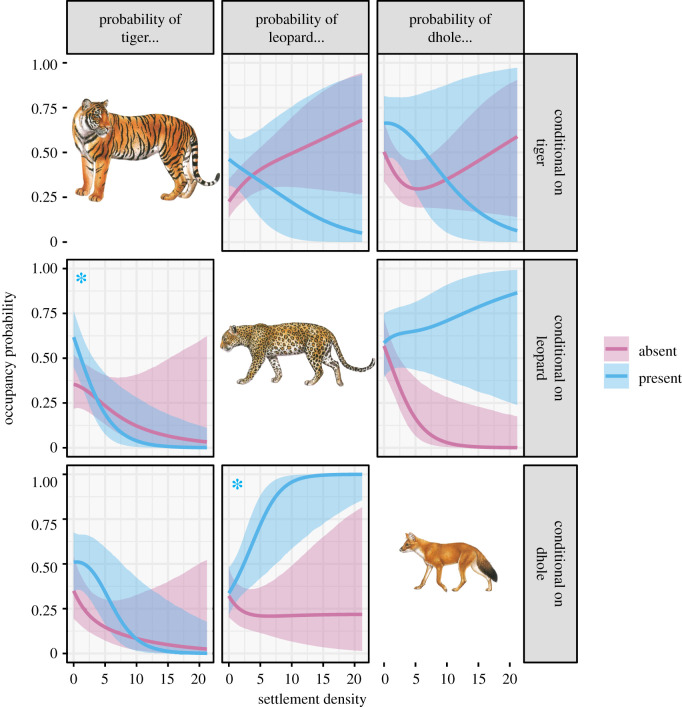
Occupancy probability of tiger, leopard and dhole conditional on the presence and absence of each of the other species along a human settlement density gradient around each camera trap. The probability of the species in each column is conditional on the presence and absence of the species in each row. The posterior means are represented by lines and 95% credible intervals by shaded ribbons. Asterisks indicate a significant relationship. Image courtesy [54]. (Online version in colour.)

Evidence that the occupancy probability of one species varied in the presence and absence of another species was apparent for only one species pair: tiger and dhole indicated by the exclusion of zero from the credible intervals of intercept parameters in the linear models ($\gamma_{0}^{\mathrm{tig}|\mathrm{dho}}=2.74[0.92-5.18]$; electronic supplementary material, appendices S8–S10). We found strong statistical support that the probability that two species occurred together varied as a function of the human settlement density within a 4 km radius of a camera station in two species pairs: tiger and leopard and leopard and dhole. The probability of occupancy of leopard as a function of settlement density varied markedly depending on whether tigers and dholes were present. At low levels of human settlement density, tigers were more likely to occupy sites if leopards were present ($\gamma_{\mathrm{Set}}^{\mathrm{tig}|\mathrm{leo}}=-1.62[-3.16--0.26]$) and occurred largely independent of dholes (figure 3, panel 7). At a higher level of human settlement density, leopards were more likely to occupy sites where dholes were also present ($\gamma_{\mathrm{Set}}^{\mathrm{leo}|\mathrm{dho}}=2.05[0.79-3.76]$) and occurred largely independent of tigers (figure 3, panel 2). We found strong evidence that tigers and leopards were likely to occupy sites together when gaur was abundant ($\gamma_{\mathrm{Gaur}}^{\mathrm{tig}|\mathrm{leo}}=2.29[0.31-4.56]$). However, there was no evidence that other prey species and human disturbance affected the interaction between species pairs (95% credible intervals straddled zero).

The probability of occupancy of dhole was highest (*ψ*_dhole_ = 0.38[0.15–0.69]) followed by leopard (*ψ*_leopard_ = 0.26[0.09–0.49]) and tiger (*ψ*_tiger_ = 0.1[0.01–0.28]). Similarly, the probability of detecting a leopard was higher (*p*_leopard_ = 0.08[0.05–0.12]) compared with tiger (*p*_tiger_ = 0.04[0.02–0.07]) and dhole (*p*_dhole_ = 0.04[0.02–0.06]).

## 4. Discussion

Our study revealed that patterns of sympatry among large carnivores in a heterogeneous landscape is mediated by settlement density and prey abundance. We provide evidence that human settlement modifies the direction and strength of interaction among carnivores [8]. We show that the multi-carnivore system is negatively affected by human settlement density at the landscape level. Spatial overlap between tigers and leopards was lower in areas with high human settlement density, whereas it was higher between leopards and dholes in similar settings. Likewise, gaur abundance positively influenced tiger and leopard interaction. Our results improve our understanding of how human activity at the site level and human infrastructure at the landscape level affect large carnivore behaviour. The above findings demonstrate that space-use patterns are likely driven by a combination of the behavioural mechanism of each species (i.e. response to environmental factors such as habitat, prey, and disturbance) and interaction with competing species [19]. This study examined interactions among globally threatened large carnivores in an understudied Himalayan landscape and provides important insights into the human–carnivore interface.

Human settlements are fragmenting natural habitats, increasingly forcing spatial interactions among large carnivores [55]. We demonstrate a clear difference in the effects of settlement density on these interactions. The strong positive interaction between the leopard–dhole pair in areas with high settlement density could be due to both species being attracted to anthropogenic resources (e.g. livestock, garbage), and that the benefits of accessing them are higher than any ensuing intraguild competition. Another plausible reason is that wild ungulates are attracted to crop fields—typically located near human settlements, which in turn attract carnivores [56]. However, large predators that share the same space and prey must, according to theory, segregate along the temporal axis to coexist [11]. Such temporal segregation seems likely given that dholes are diurnal hunters, whereas leopards are nocturnal [57].

On a cautionary note, increasing utilization of areas with high human settlement density by leopards and dholes might be an ecological trap. Leopards and dholes are notorious livestock predators [58–60] and could perhaps face fatal retaliation from affected farmers. Poignant evidence to the effect of such persecution is that dholes in Bhutan were almost entirely extirpated in the early 1980s due to widespread poisoning of livestock carcasses in retaliation to predation [61]. Consequently, an explosion of wild pig (*Sus scrofa*) populations, in turn, increased damage to crops [61]. Such trophic cascade effects due to the removal of top predators from an ecosystem are widely recognized [7]. Perhaps a reminder of the toil suffered by dholes was partially reflected in our study by the negative response (marginal occupancy) to human settlement density. Lax livestock husbandry coupled with free-range grazing fuels the growing conflict between humans and large carnivores in Bhutan [58]. Studies showed that livestock depredation by large carnivores mostly occurred in forests near the settlement where livestock was left unattended [58,60]. Thus, our finding implies that the ubiquity of human presence may benefit carnivore interaction but must be traded off against the risk of escalating conflict with humans.

Settlement density, however, had a strong negative effect on tiger–leopard interaction. This suggests that tigers and leopards may compete more directly in resource-limited areas [20] than leopards and dholes. This may result in spatial segregation [11] and/or displacement of leopards to sub-optimal habitats [62]. Studies have documented that in the presence of tigers, leopards used habitats near human settlement, consequently increasing predation on domestic livestock and ultimately conflict [58,63]. In a multi-carnivore system, our findings demonstrate the negative effect of human settlement and its consequences on interaction and habitat use. Congruent with our hypothesis, gaur abundance positively mediated tiger–leopard interaction suggesting the possibility of co-occurrence in the presence of abundant large prey. Although leopards may not directly hunt adult gaur, they are known to target calves and weaklings or scavenge on tiger kill carcasses [64]. Furthermore, it is possible that in a prey-rich environment, tigers may prefer large-bodied prey sparing smaller prey species for leopards thus segregating along the diet niche [65,66]. Our findings highlight that abundant large prey is critical for supporting large carnivore communities [67].

Although we did not find any concrete evidence of human disturbance (measured by the daily encounter rates of humans and livestock) on carnivore occupancy probability at a camera station level, growing evidence from other studies (both observational and experimental studies) suggests that large carnivores exhibit a strong fear response to humans [68–70]. Our findings rather show that human disturbance at the camera station level was negatively associated with the detection probability of all three species. Studies elsewhere show mixed results, ranging from the negative effect on carnivore detections to neutral or positive effect on habitat use [8,68,69,71]. These differences may partly be due to the spatial distribution of human presence on the landscape. For example, the human population density in western Bhutan is higher than in central, south or eastern Bhutan where the spatial interaction between the two species was low (electronic supplementary material, appendix S16).

### (a) Limitations

Observational studies such as camera trap surveys are often inadequate to infer the true processes underpinning carnivore coexistence, and hence the causal effects. Future studies could undertake studies across multiple years to infer dynamics of interspecific interactions—a pattern worth exploring in changing landscapes [72]. We acknowledge that temporal segregation might facilitate syntopy [11] but also allow coexistence with humans [73]. Moreover, study species abundance may induce heterogeneity in detection probability [74,75]. Therefore, investigating the effect of abundance on interaction could help better understand the mechanistic underpinnings of co-occupancy. Direct observation of feeding behaviour and prey choice is impossible with a camera trap survey. Our results could be better interpreted if dietary information using the scat-analysis method was available [76]. Furthermore, underlying correlates of human settlement such as poaching, roadkill and land expansion that affect carnivore co-occurrences warrant further research.

### (b) Management implications

The coexistence between humans and large carnivores is contentious [77] and in most instances, humans purportedly perceive carnivores as a threat to their lives and livelihoods resulting in conflictual responses. The polarity of human perceptions about coexisting with large carnivores has produced a mixed response and stymied conservation efforts. Management intervention aimed at protecting a single apex carnivore may need to consider the nature of interaction with other carnivores and its surrounding environments. For example, in India's Rajaji National Park, recovery of tiger population spatially displaced leopards to human-dominated habitats, the latter increased depredation on livestock and consequently suffered a decline in their numbers due to conflict and retaliation [63]. Such imbalance may jeopardize conservation efforts and induce antagonism and aversion towards large carnivores.

Conservation of multi-carnivore system would benefit from limiting the conversion of forests to other land-use types and the protection of natural habitats (irrespective of topography, e.g. rugged terrain, river network) and prey. However, as the human footprint expands rapidly, human–carnivore interaction is inevitable. Land-sharing models (where humans and wildlife share the same landscape) may need to be adopted and tailored in the context of a working landscape mosaic. Our study suggests the importance of protecting habitats adjacent to human settlements and therefore highlights the potential for conservation prioritization outside protected forest reserves. Further amending livestock husbandry practice to reduce spatial overlap between livestock and carnivores by employing guard dogs, corralling animals at night, securing overnight shelters, and supervising and guarding while grazing would be crucial to minimizing conflict [78]. Protecting crop fields by employing strategies such as electric fencing, biophysical barriers and sound repellents may help deter wild ungulates and minimize crop raids. Hunting wild ungulates for meat or in retaliation to crop damage may sever the natural food supply for carnivores [79] and inadequate wild prey may drive carnivores to increase predation of livestock as an alternative [80]. Therefore, implementing ‘collateral conservation’ measures (i.e. protecting habitat, removing snares) to protect a broad prey base may enhance carnivore conservation efforts [67].

## Data Availability

Data available via the Dryad Digital Repository: https://doi.org/10.5061/dryad.w6m905qrp [81] and sample R script via GitHub: https://github.com/ugyenpenjor1/Multispecies-Interaction-Model.
